# Assessment of the ameliorative effect of curcumin on pendimethalin-induced genetic and biochemical toxicity

**DOI:** 10.1038/s41598-022-06278-5

**Published:** 2022-02-09

**Authors:** Ali Acar, Divya Singh, Akhileshwar Kumar Srivastava

**Affiliations:** 1 Department of Medical Services and Techniques, Vocational School of Health Services, Giresun University, Giresun, Turkey; 2 Central Sericultural Research and Training Institute, Mysore, India; 3 Central Food Technological Research Institute, Mysore, India

**Keywords:** Plant sciences, Plant stress responses, Environmental sciences, Environmental chemistry

## Abstract

The present study aimed to assess the toxic effects of pendimethalin herbicide and protective role of curcumin using the *Allium* test on cytological, biochemical and physiological parameters. The effective concentration (EC_50_) of pendimethalin was determined at 12 mg/L by the root growth inhibition test as the concentration reducing the root length by 50%. The roots of *Allium cepa* L. was treated with tap water (group I), 5 mg/L curcumin (group II), 10 mg/L curcumin (group III), 12 mg/L pendimethalin (group IV), 12 mg/L pendimethalin + 5 mg/L curcumin (group V) and 12 mg/L pendimethalin + 10 mg/L curcumin (group VI). The cytological (mitotic index, chromosomal abnormalities and DNA damage), physiological (rooting percentage, root length, growth rate and weight gain) and oxidative stress (malondialdehyde level, superoxide dismutase level, catalase level and glutathione reductase level) indicators were determined after 96 h of treatment. The results revealed that pendimethalin treatment reduced rooting percentage, root length, growth rate and weight gain whereas induced chromosomal abnormalities and DNA damage in roots of *A. cepa* L. Further, pendimethalin exposure elevated malondialdehyde level followed by antioxidant enzymes. The activities of superoxide dismutase and catalase were up-regulated and glutathione reductase was down-regulated. The molecular docking supported the antioxidant enzymes activities result. However, a dose-dependent reduction of pendimethalin toxicity was observed when curcumin was supplied with pendimethalin. The maximum recovery of cytological, physiological and oxidative stress parameters was recorded at 10 mg/L concentration of curcumin. The correlation studies also revealed positive relation of curcumin with rooting percentage, root length, weight gain, mitotic activity and glutathione reductase enzyme level while an inverse correlation was observed with chromosomal abnormalities, DNA damage, superoxide dismutase and catalase enzyme activities, and lipid peroxidation indicating its protective effect.

## Introduction

The enhancement of agricultural yield is accomplished by using agrochemicals including fertilizers and pesticides to fulfill the food requirement of the population. Because the availability of agricultural land is limited and also the major threat to global food security is weeds that compete with agricultural crops for water, sunlight and nutrition, thus decreasing agricultural production^1^. Herbicides play a key role in controlling weeds and consequently contribute to global food production. In 1941, Robert Pokorny introduced the first synthetic herbicide 2,4-D. After this, several herbicides have been discovered. Although, the production of vegetables has been enhanced by the implication of herbicides, their continuous use for many years may cause various environmental issues. They may be adsorbed by the soil and influence the quality and yield of the next crop. Herbicides may pollute surface water and groundwater through irrigation, spray drift, run-off and leaching. In addition, plants may absorb stable herbicides and convert them into unwanted residues^2^. The environmental factors influence the transformation of herbicides applied in the field. The persistence of herbicides may give rise to several health-related problems. Herbicides can also affect non-targeted organisms. However, the fate of herbicides in the soil is determined by several processes including application rate, agricultural practices, crop variety, transportation, transformation, adsorption, and climatic conditions^3^. Thus, the application of persistent herbicides requires knowledge of their dissipation and movement in the soil.

Pendimethalin (*N*-(1-ethylpropyl)-2,6-dinitro-3,4-xylidine), a dinitroaniline group-selective herbicide, is widely used to control a wide variety of weeds and broadleaf plants^4^. Generally, it is applied to soil before the emergence of seedlings, planting and sometimes early post-emergence. In light sandy loam soil, pendimethalin can penetrate to root zone and exert phytotoxic effects in presence of adequate moisture^5^. Pendimethalin can inhibit cell division causes chromosomal aberrations and interfere in the formation of cell wall^6^. It also retards root and shoot growth of plants^7^. Pendimethalin is a low mobile and low volatile dinitroaniline compound having poor water solubility^8^. Previously, numerous experiments have been conducted to study the phytotoxicity of pendimethalin in different crop species such as rice^9^, cotton^10^, pigweed species, common lambsquarters, barnyardgrass^11^, paspalum cultivars^12^ and various summer season flora^13^.

It has been reported that pendimethalin and other dinitroaniline herbicides might be adsorbed and degraded in the soil^14–16^, its lipophilicity, stability and soil adsorption characteristics pose a potential risk to the environment^17,18^. Dinitroaniline compounds are also toxic to non-targeted invertebrates and aquatic organisms. In addition, dinitroaniline can form a carcinogenic compound nitrosamine that is harmful to humans^19^.

Pendimethalin can cause oxidative stress and inhibit the antioxidant system of cells^20^. The excess production of reactive oxygen species (ROS) resulting protein oxidation, cell membrane damage, inactivation of enzymes, RNA and DNA damage^21^. ROS interacts with biomolecules and damages cellular processes that result in the reduction of yield and plant growth. ROS can damage the cell membrane causing lipid peroxidation that is a highly destructive process. Lipid peroxidation may alter the structure and function of cell membranes by oxidizing polyunsaturated fatty acids (PUFAs). Impairment of PUFAs may lead to loss of fluidity and secretary functions. Malondialdehyde (MDA), an important indicator of lipid peroxidation, may be produced as a by-product of PUFA oxidation^22,23^.

Plants have numerous enzymatic and non-enzymatic defense mechanisms to overcome the impact of ROS. They accumulate different metabolites such as osmoprotectants and amino acids to assure from stress. In addition, enzymatic antioxidants are one of the most important mechanisms of plants to counter stress conditions. However, toxic compounds can perturb the activity of antioxidant enzymes such as catalase (CAT), superoxide dismutase (SOD), glutathione reductase (GR), ascorbate peroxidase (APX) and glutathione-*S*-transferase (GST) reflecting the toxicity level and plant stress tolerance capability. The protein interactions with compounds using molecular docking tool provide information about the predominant mode of interaction and binding efficacy of protein and ligand presenting protein–ligand complexes as 3D-crystal structures^24,25^. Similarly, pendimethalin herbicide may disrupt the natural structure of enzymes/proteins by interacting with their residues.

Turmeric (*Curcuma longa* L.) is a spice that has increased an interest toward both the medical/scientific and culinary communities. Turmeric is a rhizomatous herbaceous perennial plant of the ginger (*Zingiberaceae*) family^26^. Turmeric has an important component curcumin a curcuminoid as well as essential oils. Curcuminoids are phenolic chemicals derived from the roots of the *C. longa* L. and other species of *Curcuma*. Curcumin (diferuloylmethane) is a low molecular weight polyphenol that was first chemically identified in 1910. It is often regarded as the most active component, comprising about 2–8% of most turmeric formulations^27^. Curcumin (1,7-bis(4-hydroxy-3-methoxyphenyl)-1,6-heptadiene-3,5-dione), a polyphenolic compound, is a brightly yellow color pigment and the main derivative of *Curcuma* spp. Curcumin has been demonstrated to target several signaling molecules while also displaying action at the cellular level, lending evidence to its numerous health advantages^28^. It has the ability to alter Stat3 phosphorylation and DNA binding activity in human cancer cells^29^. Several in vitro and in vivo studies have shown that the therapeutic potential of curcumin does not cause side effects in animal models or humans, even at very high doses^30^. Curcumin has been linked to several therapeutic effects, including the regulation of cancer cell proliferation via different biological pathways such as apoptosis, mutagenicity, cell cycle regulation, angiogenesis, invasion, and tumorigenesis^31^. It has also antioxidant, anti-microbial, anti-parasitic and anti-inflammatory properties. It has been reported that curcumin scavenges ROS by regulating antioxidant enzyme activities in hyperglycemia^32^. All the effects of curcumin have been reported in animal models whereas no any such evidences are explored in case of plant system so far.

*Allium cepa* L. is one of the most suitable plant models for investigating the toxic effects of environmental pollutants. It is a very sensitive assay to study chromosome aberrations induced by several toxic chemicals^33,34^. The accuracy of this test is indicated by the similarity in the toxicity results revealed in different experiments using *A. cepa* L., cell culture (in vitro) and animal tests (in vivo)^35–38^. *Allium* test has also been used to explore the harmful effects of environmental pollutants on normal cell division. This test is a suitable technique to elaborate information on toxic substances induced chromosomal alterations, inhibition of mitotic activity and DNA damage with a detoxification mechanism^39,40^.

The present study investigated the effects of pendimethalin on root development (cell division and elongation kinetics) and antioxidant enzymes (SOD, CAT and GR) level as well as the interactive potential of pendimethalin molecule in *A. cepa* L. In addition, the protective role of curcumin against these effects was also assessed.

## Materials and methods

### Test organisms

The equal-sized (25–35 mm diameter, untreated) *A. cepa* L. bulbs purchased from a local market were used as the test material. *A. cepa* L. (*Amaryllidaceae*) (2n = 16) was defined using taxonomic characters and approved at the Department of Botany, Faculty of Arts and Sciences, Giresun University. Bulbs were stored in a cool and dry environment. Before application, the external scales as well as brownish base plates were removed from the bulbs without damaging the root primordia.

### Determination EC_50_ concentration

The root inhibition test was carried out by the modified method of Fiskesjö^41^. The half-maximal effective concentration (EC_50_) of pendimethalin was calculated by the *Allium* root growth inhibition test as the concentration that reduced the root length of *A. cepa* L. bulbs by 50% compared to the control group. Bulbs were treated with distilled water (negative control) and increasing concentrations of pendimethalin (by creating a group for each 1 mg/L between 1 and 20 mg/L) for 96 h at room temperature. The solutions applied to the bulb roots were renewed every 24 h. After 96 h of application, EC_50_ was calculated by the length of an average of 50 roots from 6 onions in each group. The mean root length of the control groups was considered to be 100% and the point of 50% growth point was identified as the EC_50_ dose depending on the test concentration. The EC_50_ value for pendimethalin was determined as 12 mg/L by root inhibition test and was used as the application dose in the study.

### Experimental design

The experiments based on plant were performed in accordance with international guidelines and regulations. The experiments had been comprised into in six groups. The control group was treated with tap water, application groups were treated with curcumin (5 and 10 mg/L), EC_50_ concentration of pendimethalin (12 mg/L) and their combination at 24 °C for 96 h (Table 1). The roots were exposed to the treatment solution directly in 60 × 42 mm beakers. About 40 bulbs from each group were used for physiological study and 10 bulbs were selected randomly for cytological and biochemical studies.

**Table 1:** Treatment groups.

Groups	Treatment
Group I	Tap water
Group II	5 mg/L curcumin
Group III	10 mg/L curcumin
Group IV	12 mg/L pendimethalin
Group V	12 mg/L pendimethalin + 5 mg/L curcumin
Group VI	12 mg/L pendimethalin + 10 mg/L curcumin

### Physiological parameters

About 50 bulbs from each group were used for the determination of root length based on radical formation using a millimeter ruler. The weight gain was measured using precision scales before and after treatment. The rooting percentage and relative injury rate was calculated using Eqs. (1) and (2)^42^.

1 $$ {\text{Percentage }}\,{\text{of }}\,{\text{rooting}}\left( {\text{\% }} \right) = \frac{{{\text{Rooted}}\,{\text{ Bulbs}}}}{{{\text{Total }}\,{\text{Number}}\,{\text{ of }}\,{\text{Bulbs}}}} $$

2 $$ {\text{Relative }}\,{\text{injury}}\,{\text{ rate}} = \frac{{{\text{\% Rooted }}\,{\text{bulbs}}\,{\text{ in }}\,{\text{control}} - {\text{ \% Rooted }}\,{\text{bulbs}}\,{\text{ in}}\,{\text{ each }}\,{\text{group}}}}{{{\text{\% Rooted }}\,{\text{bulbs }}\,{\text{in }}\,{\text{control}}}} $$

### Determination of chromosomal abnormalities (CAs), micronucleus (MN) and mitotic index (MI)

The root tips were fixed for 2 h in Clarke solution (ethanol, glacial acetic acid, 3:1) followed by 96% ethanol for 15 min. Then samples were processed in 70% ethanol at 4 °C. For cytological studies, the roots were hydrolyzed in 1 N HCl for 17 min at 60 °C, incubated with 45% acetic acid for 30 min and stained for 24 h in acetocarmine. Preparations of mitotic cells were analyzed under a binocular microscope at ×500 magnification^43^. The evaluation of the MN involvement has been carried out according to Fenech et al^44^.

For each group, 10 slides were prepared from randomly selected bulbs, 1000 cells for MN and CAs frequency and 10,000 cells for MI were counted in each slide (Eq. 3).

3 $$ {\text{Mitotic }}\,{\text{Index }}\left( {\text{\% }} \right) = \frac{{{\text{Number }}\,{\text{of }}\,{\text{mitotic }}\,{\text{cells}}}}{{{\text{Total}}\,{\text{ number }}\,{\text{of }}\,{\text{cells}}}} $$

### Comet assay

Comet assay (alkaline single-cell gel electrophoresis) was performed according to the modified method of Tice et al*.*^45^ The nuclei were isolated from fresh root tips in 600 µL ice-cold nuclear isolation buffer (400 mM 6H_2_O–MgCl_2_, 0.5% w/v Triton X-100, 0.4 M Tris, pH 7.5) using a petri dish with a razor blade and root tips were centrifuged at 1200 rpm for 7 min after being passed through a nylon mesh filter. A mixture of 1:1 nuclear suspension with 1% low melting point (LMPA) in phosphate-buffered saline (PBS) was placed on a pre-coated slide with 1% normal melting point agarose (NMPA). Electrophoresis was performed in chilled electrophoresis buffer at 0.7 V/cm at 4 °C (20 V, 300 mA) for 15 min using a power supply. Slides were rinsed three times with filtered water and neutralized with Tris buffer (0.4 M Tris, pH 7.5). The nuclei were stained for 5 min with ethidium bromide after immersion in cold water for 5 min. The preparations were washed with cold water and eliminated residual stain and coverslip sealed. These steps were taken with low light to avoid DNA degradation and examined with a fluorescence microscope. Comets were analyzed using Comet Assay software version 1.2.3b^46^. About 100 cells per slide were analyzed for DNA damage. The extent of DNA damage was scored from 0 to 4 depending upon the level of DNA damage. The cells were classified into five categories based on tail DNA (%): 0—undamaged (0–2%), 1—low damage (< 2–25%), 2—moderate damage (< 25–45%), 3—high damage (< 45–70%) and 4—extreme damage (< 70%)^47^. The total DNA damage per sample, expressed as arbitrary units, was calculated using Eq. (4).

4 $$ {\text{Arbitrary }}\,{\text{unit}} = \mathop \sum \limits_{i = 0}^{4} N_{i} x_{i} $$

[*i*: degree of damage (0, 1, 2, 3, 4), *N*_*i*_*:* the number of cells in *i* degree].

### Evaluation of antimutagenic effects

The antimutagenic effect of curcumin was calculated for each slide by Eq. (5) and mean ± SD (standard deviation) values were calculated^48^. In order to assess the antimutagenic effect, chromosomal abnormalities (CAs) and the arbitrary unit of the comet assay was evaluated.

5 $$ {\text{Mutagenicity }}\,{\text{inhibition }}\left( {\text{\% }} \right) = \frac{{{\text{Pendimethalin }}\,{\text{Group }}\,{\text{Damage }}\left( {\text{\% }} \right) - {\text{Pendimethalin }}\,{\text{with }}\,{\text{Curcumin}}\,{\text{ Group}}\,{\text{ Damage }}\left( {\text{\% }} \right)}}{{{\text{Pendimethalin}}\,{\text{ Group}}\,{\text{ Damage }}\left( {\text{\% }} \right) - {\text{ Control }}\,{\text{Group }}\,{\text{Damage }}\left( {\text{\% }} \right)}}{ } \times { }100 $$

### Lipid peroxidation

Lipid peroxidation was evaluated by measuring the quantity of MDA according to the method of Ünyayar et al*.*^49^ Approximately 0.5 g of root tissue were homogenized with 5% trichloroacetic acid (TCA) and centrifuged at 12,000 rpm at 24 °C for 15 min. The supernatant, TCA solution (20%) and thiobarbituric acid (0.5%) has been transferred to the new tube and incubated at 96 °C for 25 min. The tubes were taken into the ice bath and centrifuged at 10,000 rpm for 5 min. The absorbance was recorded at 532 nm, the extinction coefficient was 155 mM/cm has been used to determine the quantity of MDA content.

### Antioxidant enzyme assays

#### Superoxide dismutase

SOD level was assessed according to the method of Beauchamp and Fridovich^50^. About 0.5 g of roots was homogenized in 5 mL of 50 mM (pH 7.8) chilled sodium phosphate buffer. The homogenates were centrifuged at 10,500 rpm for 20 min and the supernatant was used for the enzyme assay. The reaction mixture containing 1.5 mL 0.05 M sodium phosphate buffer (pH 7.8), 0.3 mL 130 mM methionine, 0.3 mL 0.1 mM EDTA-Na_2_,0.3 mL 750 μM nitroblue tetrazolium chloride (NBT), 0.3 mL of 20 μM riboflavin, 0.01 mL of 4% (w/v) insoluble polyvinylpyrrolidone, 0.01 mL of enzyme extract, and 0.28 mL of deionized water was prepared. The reaction began with putting the tubes under two 15 W fluorescent lamps for 10 min and ending by keeping the tubes in the dark for 10 min. Absorbance was measured at 560 nm and a unit SOD enzyme activity was determined as the amount of SOD enzyme required for 50% inhibition of NBT reduction under treatment conditions.

#### Catalase

CAT activity was determined by using the method of Beers and Sizer^51^. The reaction mixture containing 2.8 mL of 0.3 mL 0.1 M H_2_O_2_, 1 mL deionized water and 200 mM sodium phosphate (1.5 mL) was formulated just before use to evaluate the reaction combination. The reaction was triggered by adding 0.2 mL of supernatant and CAT activity was measured by monitoring the absorbance decrease (240 nm) as a result of H_2_O_2_ consumption. Units of CAT-activity were determined by units per minute per g fresh weight; one unit of CAT-activity was defined for change of 0.1 at an absorbance of 240 nm and values are taken from the measurements of three independent samples and expressed as OD_240nm_/min g FW.

#### Glutathione reductase

Glutathione reductase (GR) levels were determined by the modified method of Carlberg and Mannervik^52^. The root tips (0.5 g) were homogenized in 0.2 M EDTA (pH 4.7). The GR level was measured in a 2 mL reaction mixture containing 1 M oxidized glutathione (GSSG), 0.1 mM nicotinamide adenine dinucleotide phosphate (NADPH), 0.05 M potassium phosphate buffer (pH 7.0) and 3 mM EDTA. The supernatant absorbances were recorded at 340 nm and values are taken from the measurements of three independent samples. The levels of GR were expressed as μmol NADPH/min.g FW.

#### Molecular docking

Molecular docking was performed to analyze molecular interactions of pendimethalin with antioxidant enzymes (superoxide dismutase, catalase and glutathione reductase) and DNA molecules. The crystallographic 3D structure of the SOD (PDB ID: 1ba9)^53^, CAT (PDB ID: 5gkn)^54^, GR (PDB ID: 2hqm)^55^, B-DNA dodecamer (PDB ID: 1bna)^56^, B-DNA dodecamer d (PDB ID: 195d)^57^ and DNA (PDB ID: 1cp8)^58^ molecules were obtained from the protein data bank. The 3D structure of pendimethalin (PubChem CID: 38479) was retrieved from PubChem. Enzymes and DNA molecules were prepared using Biovia Discovery Studio 2020 Client for docking. During the preparation process, the active sites of enzymes were determined by removing the water molecules and co-crystal ligands, further polar hydrogen atoms were added to enzymes. Energy minimization of enzymes was done with Gromos 43B1 using Swiss-PdbViewer (v.4.1.0)^59^ software whereas energy minimization of the 3D structure of pendimethalin was accomplished with the uff-force field employing Open Babel v.2.4.0 software^60^. The molecular docking process was started with the grid box containing the active sites of enzymes and the entire structure for DNA. Then docking was performed using Autodock 4.2.6 software^61^ based on Lamarckian genetic algorithm (LGA) and the LGA was run for 10 runs with an initial population size of 150 individuals for both enzymes and DNA. The docking analysis and 3D visualizations were performed with Biovia Discovery Studio 2020 Client.

#### Dose–response effects of curcumin

The evaluation of the dose–response effects of curcumin against pendimethalin toxicity was performed by calculating the percentage curative effect of curcumin against the changes caused by pendimethalin toxicity in all parameters. The recovery percentage caused by curcumin in the calculation was calculated by proportioning with the pendimethalin application group and control group data. For this, Eq. (6) was used and evaluated with the logarithmic values of the doses^62^.

6 $$ {\text{Recovery}}\,{\text{Effect}}\,{\text{of}}\,{\text{Curcumin}}\left( {\text{\% }} \right) = \frac{{{\text{Pendimethalin }}\,{\text{with }}\,{\text{Curcumin }}\,{\text{Group }}\,{\text{Parameter}} - {\text{Pendimethalin }}\,{\text{Group}}\,{\text{ Parameter}}}}{{{\text{Control}}\,{\text{ Group }}\,{\text{Parameter}} - {\text{ Pendimethalin}}\,{\text{ Group }}\,{\text{Parameter}}}}{\text{ x }}100 $$

### Data analysis

Root elongation kinetics was characterized using logistic, log-logistic, Gompertz and Weibull models. The selection of the model was based on the best fit in Bayesian Information Criterion (BIC) and Akaike information criterion (AIC). The free parameters namely maximum growth (A), growth rate (µ) and length of lag phase (λ) shared by models were employed as descriptors of the kinetics. The functions of “tidyverse” and “drc” packages for R programming language were applied in root elongation kinetics^63^.

SPSS Statistics v22.0 (IBM Corp., USA, 2015) package program was used to perform statistical analyzes. Data were expressed as mean ± SD (standard deviation) in the tables and mean ± SEM (standard error of means) in the graphs. The statistical significance between the means was determined by the method of One-way ANOVA and Duncan's test and the *p* < 0.05 was deemed statistically significant. Since there were two independent variables in the analysis of mutagenicity inhibitions, independent samples t-test was used and *p* < 0.05 was deemed statistically significant.

## Results

### Effect of pendimethalin on root growth and protective role of curcumin

The effects of pendimethalin were evaluated on root length, rooting percentage, injury rate and weight gain (Table 2). About 100% rooting was recorded in group I (control), group II (5 mg/L curcumin) and group III (10 mg/L curcumin). However, the application of 12 mg/L pendimethalin (group IV) had decreased the rooting percentage to 30%. Further, combination of pendimethalin with curcumin in group V (12 mg/L pendimethalin + 5 mg/L curcumin) and group VI (12 g/L pendimethalin + 10 mg/L curcumin) had recovered rooting percentage to 47.50% and 57.50% respectively.

**Table 2:** Effect of pendimethalin and curcumin on rooting percentage and root length.

Groups	Rooting percentage (%)	Mean root length	Relative injury rate
Group I	97.50	16.92 ± 3.49^a^	–
Group II	100	17.15 ± 3.59^a^	–
Group III	100	17.30 ± 3.06^a^	–
Group IV	30.00	8.45 ± 2.53^c^	0.69
Group V	47.50	10.21 ± 2.64^bc^	0.51
Group VI	57.50	12.22 ± 2.77^b^	0.41

Group I: tap water (control), Group II: 5 mg/L curcumin, Group III: 10 mg/L curcumin, Group IV: 12 mg/L pendimethalin, Group V: 12 mg/L pendimethalin + 5 mg/L curcumin, Group VI: 12 mg/L pendimethalin + 10 mg/L curcumin. Data are shown as mean ± standard deviation (SD). The averages shown with different letters in the same column are statistically significant (*P* < 0.05).

The relative injury rate represented the severity of the damage. The highest relative injury rate was observed in group IV (0.69). The relative injury rate was low in group VI (0.41). A concentration-dependent improvement in relative injury rate was recorded due to the application of curcumin at different concentrations.

The toxicity of pendimethalin is indicated by retarded root growth. After 96 h of treatment, the mean root length was 16.92, 17.15 and 17.30 cm in group I, group II and group III respectively. About 50% reduction in root growth (8.45 cm) was recorded in pendimethalin-treated roots in group IV. However, the application of curcumin at different doses recovered root growth to 10.21 and 12.22 cm in group V and group VI respectively.

Table 3 shows the effects of pendimethalin and curcumin on weight gain. The highest weight gain was observed in group III (10.78 g) followed by group I (10.72 g) and group II (10.26 g). Pendimethalin treatment decreased the weight gain to 3.09 g in group IV. Further, the application of curcumin recovered weight gain to 4.92 and 6.37 g in group V and group VI respectively.

**Table 3:** Effect of pendimethalin and curcumin on weight gain.

Groups	Initial weight	Final weight	Weight gain (g)
Group I	12.95 ± 2.09^d^	23.67 ± 2.98^a^	10.72 ± 3.47^a^
Group II	12.16 ± 2.32^d^	22.42 ± 2.70^a^	10.26 ± 2.72^a^
Group III	12.68 ± 2.55^d^	23.46 ± 2.48^a^	10.78 ± 1.34^a^
Group IV	11.65 ± 1.75^d^	14.74 ± 2.59^ cd^	3.09 ± 1.73^c^
Group V	11.36 ± 1.41^d^	16.28 ± 2.15^bc^	4.92 ± 1.54^bc^
Group VI	11.50 ± 1.70^d^	17.87 ± 2.62^b^	6.37 ± 1.50^b^

Group I: tap water (control), Group II: 5 mg/L curcumin, Group III: 10 mg/L curcumin, Group IV: 12 mg/L pendimethalin, Group V: 12 mg/L pendimethalin + 5 mg/L curcumin, Group VI: 12 mg/L pendimethalin + 10 mg/L curcumin. Data are shown as mean ± standard deviation (SD). The averages shown with different letters in the same column are statistically significant (*P* < 0.05).

**Figure 1:**
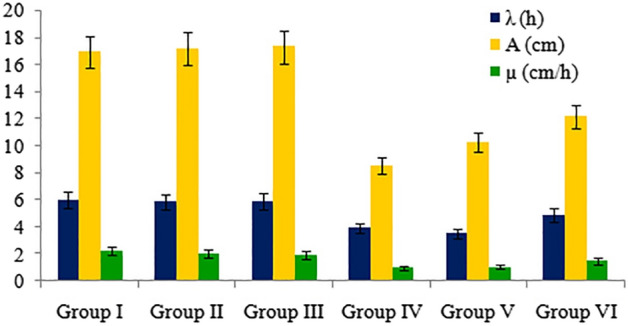
Effects of pendimethalin and curcumin treatments on root elongation kinetics.

The kinetics of root elongation revealed significant decreases in root length (A), lag phase (λ) and growth rate (µ) of pendimethalin-treated roots in comparison to control (Fig. 1). No significant difference in λ and A of group I, group II and group III was observed. However, a significant difference in µ was noted in all groups.

### Impact of pendimethalin on mitotic index and chromosomal abnormalities and protective role of curcumin

The cytogenetic effects of pendimethalin application and the protective role of curcumin were investigated by mitotic index (MI), chromosomal aberrations (CAs) and micronucleus (MN) formation. According to the data in Table 4, there was no significant difference among the MI rate of group I (9.09%), group II (9.14%) and group III (9.34%). The lowest MI (4.67%) was observed in group IV treated roots. However, a concentration-dependent increase in MI rate with the administration of curcumin (5 mg/L and 10 mg/L) and pendimethalin was recorded in group V (6.00%) and group VI (6.89%).

**Table 4:** Effects of pendimethalin on MN formation and Mitotic index (MI) and protective role of curcumin.

Groups	MN formation	Mitotic index (MI)	Mitotic index rate (%)
Group I	0.50 ± 0.85^d^	908.70 ± 36.08^a^	9.09 ± 0.36^a^
Group II	0.00 ± 0.00^d^	914.20 ± 49.92^a^	9.14 ± 0.50^a^
Group III	0.00 ± 0.00^d^	933.90 ± 46.40^a^	9.34 ± 0.46^a^
Group IV	59.30 ± 13.78^a^	467.30 ± 51.70^d^	4.67 ± 0.52^d^
Group V	42.80 ± 14.97^b^	599.80 ± 30.66^c^	6.00 ± 0.31^c^
Group VI	29.20 ± 8.47^c^	689.30 ± 40.88^b^	6.89 ± 0.41^b^

Group I: tap water (control), Group II: 5 mg/L curcumin, Group III: 10 mg/L curcumin, Group IV: 12 mg/L pendimethalin, Group V: 12 mg/L pendimethalin + 5 mg/L curcumin, Group VI: 12 mg/L pendimethalin + 10 mg/L curcumin. Data are shown as mean ± standard deviation (SD). The averages shown with different letters in the same column are statistically significant (*P* < 0.05).

**Figure 2:**
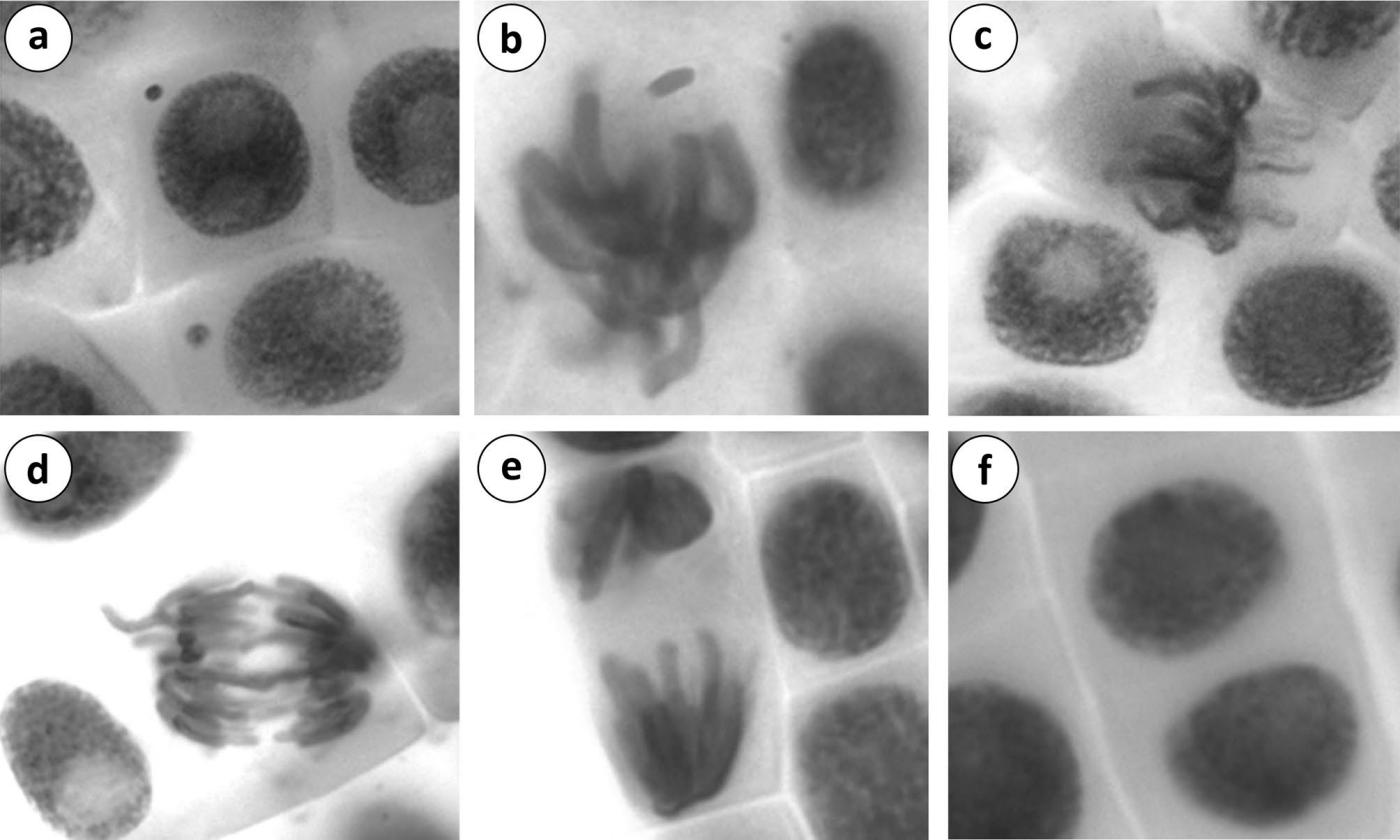
CAs and MN formations induced by pendimethalin (**a**: MN, **b**: fragment, **c**: sticky chromosome, **d**: bridge and vagrant chromosome, **e**: unequal distribution of chromatin, **f**: binucleated cell).

Different types of chromosomal abnormalities were shown in Table 5. A few abnormal cells were reported in control (group I) showing fragment, sticky chromosome and micronucleus formation. No chromosomal abnormalities were noted in roots in group II and group III treated with 5 mg/L and 10 mg/L curcumin, respectively. There was no significant difference in chromosomal abnormalities observed in group I, group II and group III. A range of chromosomal abnormalities such as fragment, sticky chromosome, bridge, binucleated cells, vagrant chromosome, unequal distribution of chromatin and micronucleus (Tables 4, 5) were recorded (Fig. 2). In addition, micronucleus formation usually indicated chromosomal instability and genotoxic effects. The frequency of chromosomal abnormalities was high in group IV treated root with pendimethalin. Administration of curcumin at 5 mg/L and 10 mg/L in group V and group VI decreased chromosomal abnormalities induced by pendimethalin. This dose-dependent decline in chromosomal abnormalities indicated the antimutagenic effect of curcumin. Mutagenicity inhibition was ascertained as 27.91% and 46.66% in group V and group VI respectively. The results indicated that chromosomal abnormalities were decreased with increasing mutagenicity inhibition.

**Table 5:** Frequencies of chromosomal abnormalities caused by pendimethalin and the protective role of curcumin.

	Group I	Group II	Group II	Group IV	Group V	Group VI
FRG	0.70 ± 0.67^d^	0.00 ± 0.00^d^	0.00 ± 0.00^d^	85.70 ± 17.80^a^	61.70 ± 16.23^b^	50.30 ± 10.51^c^
SC	0.20 ± 0.42^d^	0.00 ± 0.00^d^	0.00 ± 0.00^d^	50.30 ± 10.06^a^	38.50 ± 10.24^b^	28.90 ± 9.49^c^
B	0.00 ± 0.00^d^	0.00 ± 0.00^d^	0.00 ± 0.00^d^	41.10 ± 11.81^a^	32.50 ± 12.38^b^	22.70 ± 10.79^c^
UDC	0.00 ± 0.00^d^	0.00 ± 0.00^d^	0.00 ± 0.00^d^	30.30 ± 9.29^a^	21.60 ± 6.72^b^	14.70 ± 5.72^c^
BC	0.00 ± 0.00^d^	0.00 ± 0.00^d^	0.00 ± 0.00^d^	24.80 ± 9.38^a^	15.30 ± 4.62^b^	10.40 ± 5.42^c^
VC	0.00 ± 0.00^d^	0.00 ± 0.00^d^	0.00 ± 0.00^d^	19.90 ± 7.34^a^	12.40 ± 7.73^b^	7.90 ± 5.04^c^
Total CAs	0.90 ± 0.88^d^	0.00 ± 0.00^d^	0.00 ± 0.00^d^	252.10 ± 39.69^a^	182.00 ± 37.53^b^	134.90 ± 24.98^c^
CAs (%)	0.09 ± 0.08^d^	0.00 ± 0.00^d^	0.00 ± 0.00^d^	25.10 ± 3.97^a^	18.20 ± 3.75^b^	13.49 ± 2.50^c^
Mutagenicity inhibition(%)	–	–	–	–	27.91 ± 9.33^b^	46.66 ± 5.56^a^

Group I: tap water (control), Group II: 5 mg/L curcumin, Group III: 10 mg/L curcumin, Group IV: 12 mg/L pendimethalin, Group V: 12 mg/L pendimethalin + 5 mg/L curcumin, Group VI: 12 mg/L pendimethalin + 10 mg/L curcumin. Data are shown as mean ± standard deviation (SD). The averages shown with different letters in the same line are statistically significant (P < 0.05). FRG: Fragment, SC: Sticky chromosome, B: Bridge, UDC: Unequal distribution of chromatin, BC: binucleated cell, VC: vagrant chromosome.

**Figure 3:**
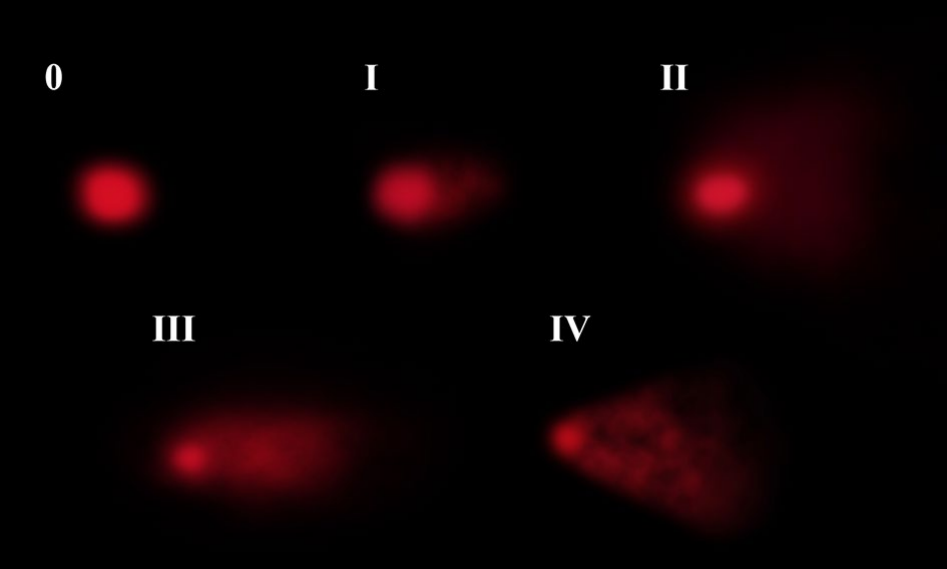
Effects of pendimethalin on DNA in *A. cepa* root tip cells nuclei (**0**: < 2% tail percentage, **I**: 2–25% tail percentage, **II**: > 25–45% tail percentage, **III**: > 45–70% tail percentage, **IV**: > 70% tail percentage).

DNA damage induced by pendimethalin in the roots of *A. cepa* L. and protecting role of curcumin were determined using single-cell gel electrophoresis test with three scales (head DNA, tail DNA and arbitrary unit) (Table 6). In the calculation of arbitrary units, cells were analyzed by classifying them into five categories (Fig. 3) according to tail DNA. There was no significant difference in head DNA (%), tail DNA (%) and arbitrary unit between control (group I) and curcumin alone applied groups (group II and group III). However, pendimethalin treatment (group IV) decreased head DNA percentage to 32.43, increased tail DNA percentage to 67.57% and arbitrary unit to 291.90. Further, administration of different doses of curcumin with pendimethalin in group V and group VI reduced the tail DNA percentage and arbitrary unit whereas increased head DNA percentage.

**Table 6:** Detection of the effects of pendimethalin and curcumin applications on DNA by comet assay.

Groups	Head DNA (%)	Tail DNA (%)	Arbitrary unit	Mutagenicity inhibition (%)
Group I	98.18 ± 1.07^a^	1.82 ± 1.07^d^	10.80 ± 0.87^d^	–
Group II	98.78 ± 1.12^a^	1.22 ± 1.12^d^	9.70 ± 1.04^d^	–
Group III	98.54 ± 1.15^a^	1.46 ± 1.15^d^	8.90 ± 1.18^d^	–
Group IV	32.43 ± 1.89^d^	67.57 ± 1.89^a^	291.90 ± 7.30^a^	–
Group V	49.14 ± 2.43^c^	50.86 ± 2.43^b^	222.80 ± 5.68^b^	24.58 ± 2.16^b^
Group VI	64.09 ± 3.60^b^	35.91 ± 3.60^c^	193.20 ± 4.08^c^	35.13 ± 1.88^a^

Group I: tap water (control), Group II: 5 mg/L curcumin, Group III: 10 mg/L curcumin, Group IV: 12 mg/L pendimethalin, Group V: 12 mg/L pendimethalin + 5 mg/L curcumin, Group VI: 12 mg/L pendimethalin + 10 mg/L curcumin. Data are shown as mean ± standard error of means (SEM). The averages shown with different letters in the same column are statistically significant (*P* < 0.05).

### Effect of pendimethalin on lipid peroxidation and antioxidant enzymes and protective role of curcumin

**Figure 4:**
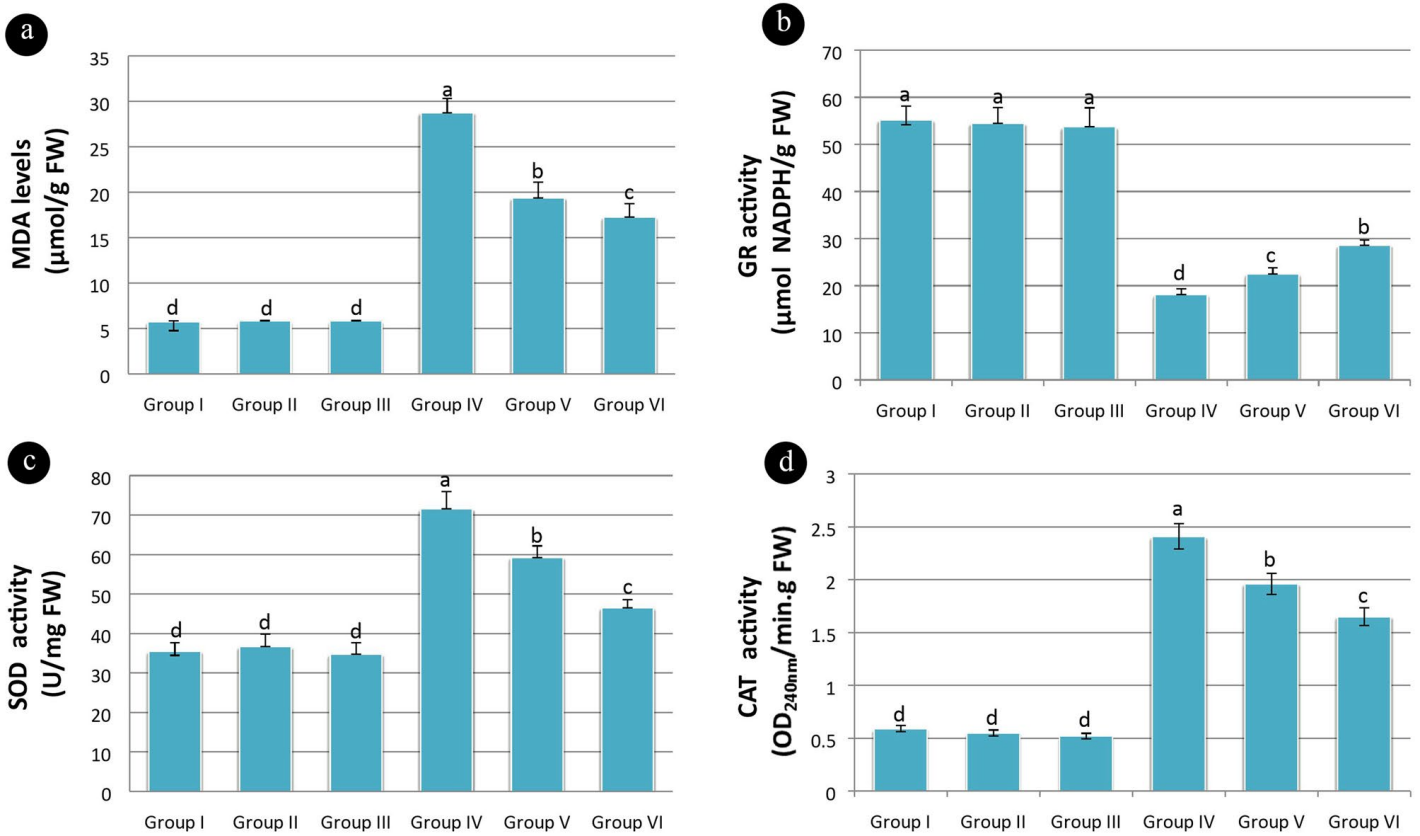
Effects of pendimethalin and curcumin applications on lipid peroxidation and antioxidant enzyme activities (**a**: MDA levels, **b**: GR levels, **c**: SOD levels **d**: CAT levels) [Data were shown as mean ± SEM. The averages shown with different letters in each graph are statistically significant (*P* < 0.05)].

MDA level is an important indicator of lipid peroxidation. MDA levels in group I, group II and group III was not statistically significant and were recorded as 5.75, 5.83 and 5.86 µmol/g FW respectively. However, the application of pendimethalin (group IV) increased MDA level (28.75 µmol/g FW). The application of curcumin along with pendimethalin in group V and VI recovered MDA levels as 19.36 and 17.25 µmol/g FW respectively. A significant difference in MDA level was observed among group IV, group V and group VI (Fig. 4a).

The antioxidant enzymes activities mitigate and repair ROS damage in plants. GR maintains the level of reduced glutathione in the cell. The reduced form of glutathione plays important role in the control of ROS. GR levels in group I, group II and group III was insignificant and noted as 55.16, 54.45 and 53.75 µmol NADPH/g FW respectively. GR levels in group IV, group V and group VI were significantly different from the above groups and recorded as 18.15, 22.48 and 28.56 µmol NADPH/g FW respectively (Fig. 4b).

SOD activity was affected by the production of superoxide radicals. SOD levels in control and curcumin alone applied groups were recorded as 35.46, 36.65 and 34.72 U/mg FW respectively. SOD levels in other groups were significantly higher than in the control. SOD levels in group IV, group V and group VI were recorded as 71.56, 59.25 and 46.5 U/mg FW respectively (Fig. 4c).

CAT neutralizes the generated hydrogen peroxide in plant cells by detoxifying it into water and oxygen. CAT levels in group I (0.59 OD_240nm_/min.gFW), group II (0.55 OD_240nm_/min.g FW) and group III (0.52 OD_240nm_/min.g FW) were insignificant. However, CAT levels in group IV (2.41 OD_240nm_/min^.^g FW), group V (1.96 OD_240nm_/min.gFW) and group VI (1.65 OD_240nm_/min^.^g FW) were significantly different from group I, group II and group III (Fig. 4d).

**Figure 5:**
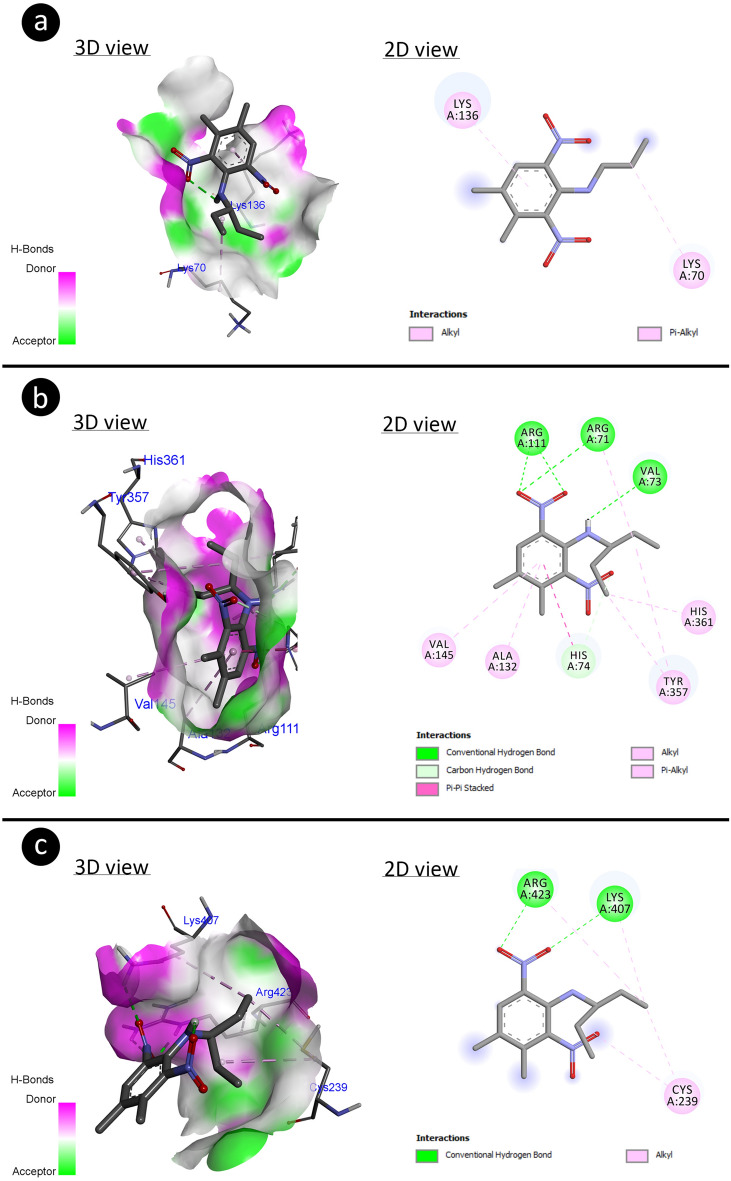
Potential molecular interactions of pendimethalin with antioxidant enzyme residues with different modes including H-bond (**a**: SOD-pendimethalin, **b**: CAT-pendimethalin, **c**: GR-pendimethalin).

**Figure 6:**
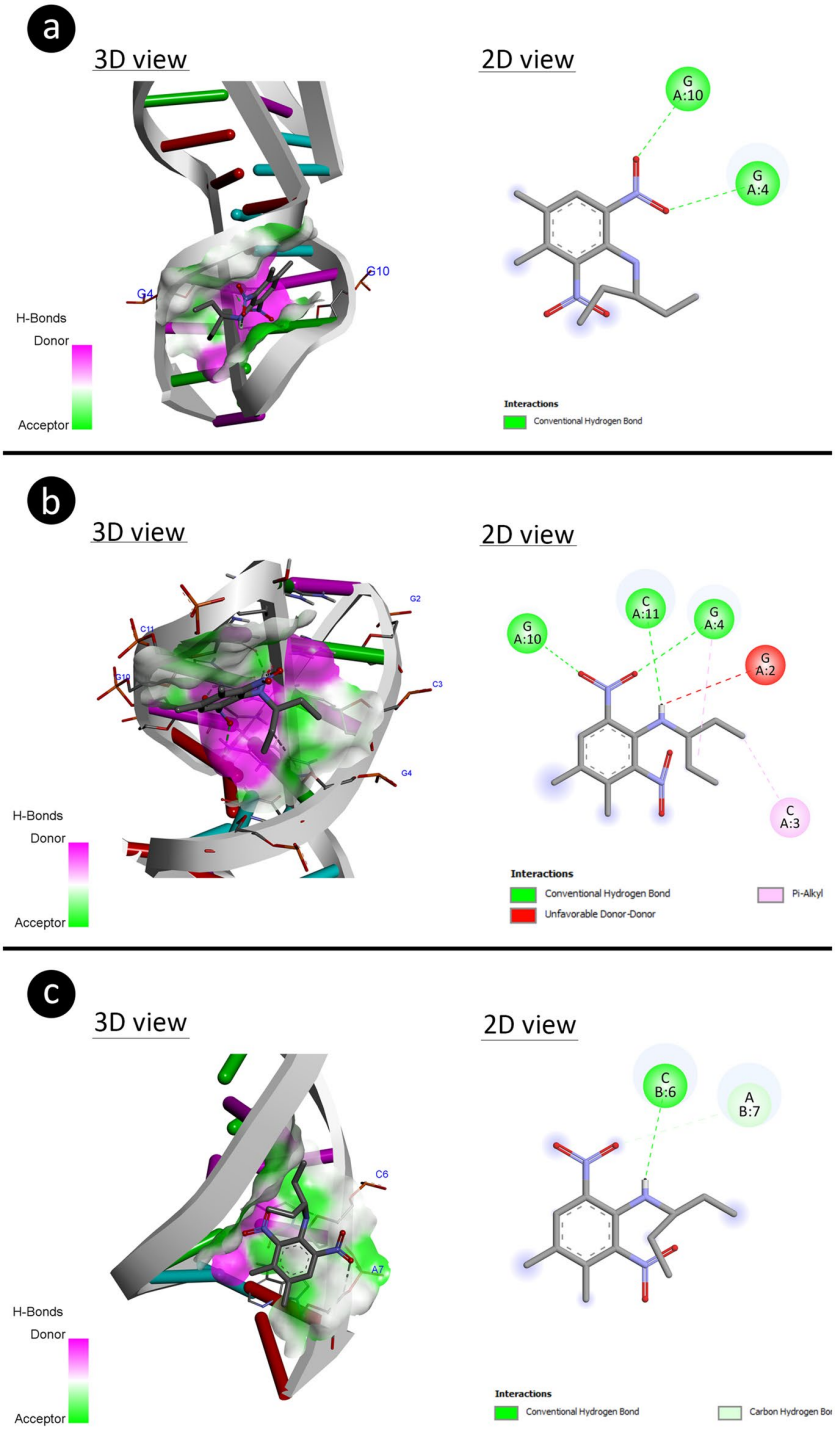
The 3D structural interaction of pendimethalin-DNA through different patterns for potential DNA disruption (**a**: 1BNA-pendimethalin, **b**: 195D-pendimethalin, **c**: 1CP8-pendimethalin).

In addition, the results of molecular docking based on binding energy revealed that pendimethalin has capable to interact with antioxidant enzymes (Fig. 5) as well as DNA molecules (Fig. 6).

Table 7 explains the binding potential of pendimethalin with different antioxidant enzymes. The pendimethalin made hydrophobic interaction to residues (LYS70 and LYS136) of SOD with binding energy (− 1.83 kcal/mol) and inhibition constant (Ki: 45.34 mM). The residues (VAL73, ARG71, ARG111 (×2), HIS74) of CAT have interacted with pendimethalin through H-bond and other residues ARG71, ALA132, VAL145, TRY357 (×2), HIS361: made hydrophobic interaction which involved binding energy (− 8.23 kcal/mol) and inhibition constant (930.77 nM). The hydrophobic interaction by residues: CYS239 (×2), LYS407, ARG423 and H-bond interaction by residues LYS407 and ARG423 (×2) were participated in making complexation with GR by involving binding energy (− 3.15 kcal/mol) and inhibition constant (4.89 mM).

**Table 7:** Energy involved during molecular interactions of pendimethalin with antioxidant enzymes.

Antioxidant enzymes	Free energy of binding (kcal/mol)	Inhibition constant (*K*_*i*_)	Hydrogen bond interactions	Hydrophobic interactions
SOD	− 1.83	45.34 mM	–	LYS70 LYS136
CAT	− 8.23	930.77 nM	VAL73 ARG71 ARG111 (×2) HIS74	ARG71 ALA132 VAL145 TRY357 (×2) HIS361
GR	− 3.15	4.89 mM	LYS407 ARG423 (×2)	CYS239 (×2) LYS407 ARG423

The B-DNA Dodecamer(1BNA) had made complex with pendimethalin through its bases (G4 and G10) of nucleic acid interaction using binding energy (− 3.97 kcal) and inhibition constant (1.24 mM). The pendimethalin has integrated with bases (G4, G10, and C11) of B-DNA Dodecamer D (195D) dissipating binding energy (− 4.71 kcal/mol) with inhibition constant (Ki 354.55 μM). The bases (C6 and A7) of DNA (1CP8) have complexed with pendimethalin by involving binding energy (− 4.34 kcal/mol) with inhibition constant (Ki 7.81 mM). The above explanation about the interactive efficiencies of pendimethalin toward DNA has been tabulated in Table 8.

**Table 8:** The binding energy of the interacted pendimethalin with DNA molecules.

DNA molecule	DNA sequence	Free energy of binding (kcal/mol)	Inhibition constant (*K*_*i*_)	Interacting nucleic acids
B-DNA dodecamer (1BNA)	5′-CGCGAATTCGCG-3′	− 3.97	1.24 mM	G4 G10
B-DNA Dodecamer D (195D)	5′-CGCGTTAACGCG-3′	− 4.71	354.55 μM	G4 G10 C11
DNA (1CP8)	5′-TTGGCCAA-3′	− 4.34	7.81 mM	C6 A7

### Dose-depended protective effects of curcumin and its correlation with physiological, genetical and antioxidant enzymes

**Figure 7:**
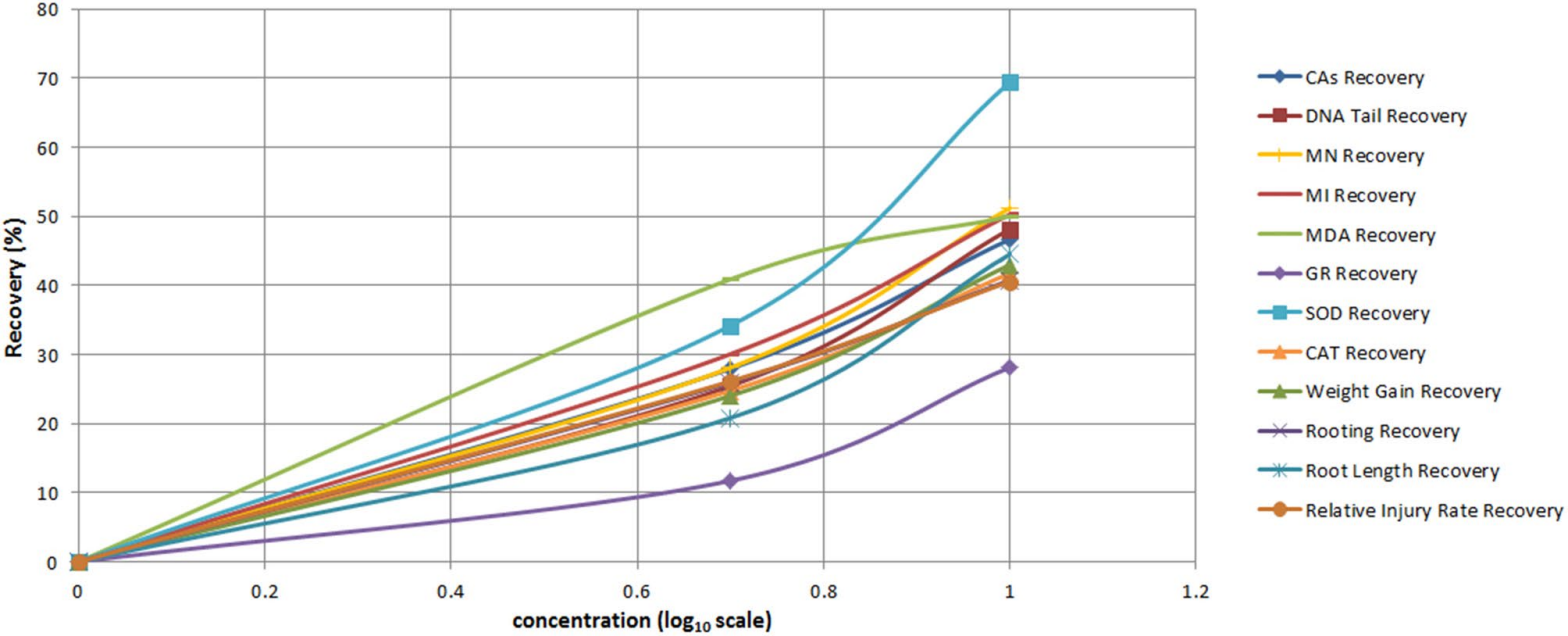
Dose–response protection curves of curcumin against pendimethalin toxicity.

The dose-dependent protecting effects of curcumin against pendimethalin has been shown in Fig. 7. A concentration-dependent recovery in all parameters was observed after synergistic use of curcumin with pendimethalin. The highest protecting effect of curcumin was observed at 10 mg/L concentration. This concentration of curcumin recovered physiological parameters from 40.74 to 42.99%, genetic parameters 46.66–51.19%, antioxidant enzymes levels along lipid peroxidation from 28.13 to 69.42%.The highest recovery (69.42%) was noted in SOD enzyme level. The results indicated a direct or indirect relation among all the parameters.

**Figure 8:**
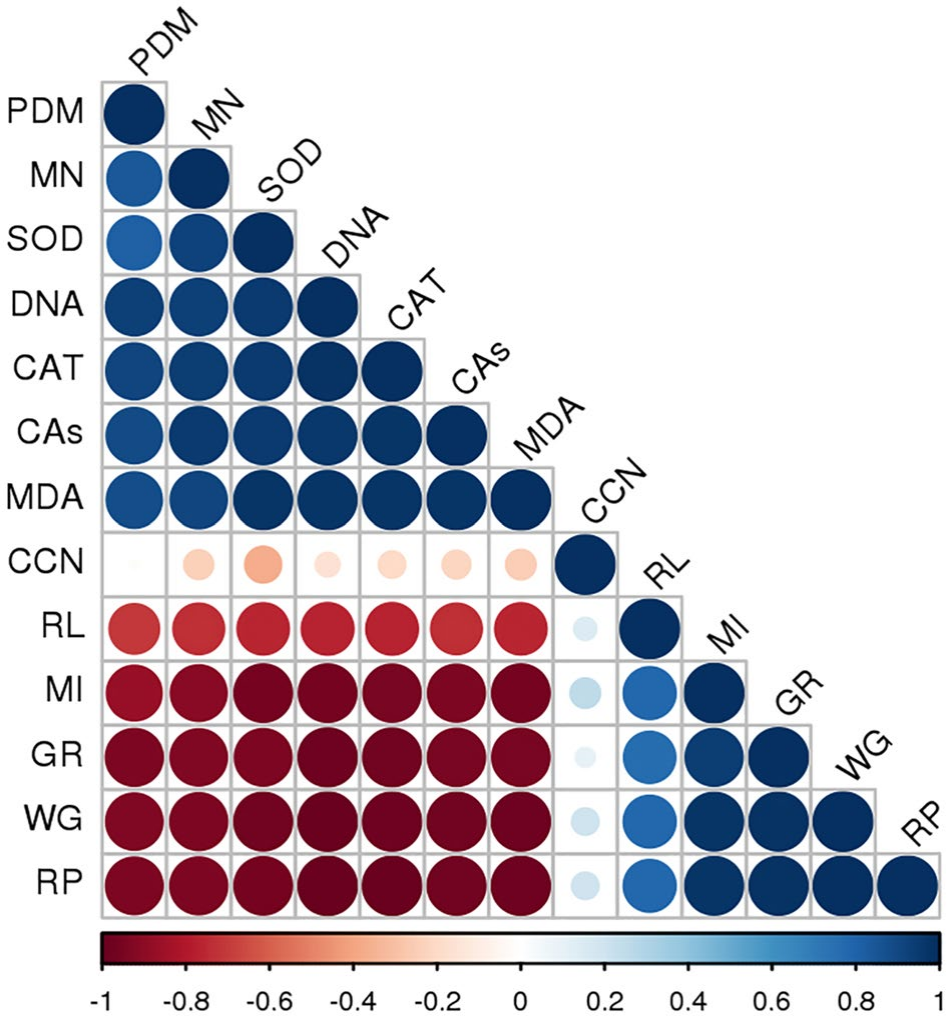
Correlation of pendimethalin and curcumin with cytological, physiological and biochemical analysis (**PDM**: pendimethalin, **MN**: micronucleus, **SOD**: superoxide dismutase, **DNA**: DNA arbitrary unit bridge, **CAT**: catalase, **CAs**: chromosomal abnormalities, **MDA**: malondialdehyde, **CCN**: curcumin, **RL**: root length, **MI**: mitotic index, **GR**: glutathione reductase, **WG**: weight gain, **RP**: rooting percentage. Pearson correlation analysis (two-sided) was performed and visualized with Rstudio software. Positive correlations are shown in blue and negative correlations in red. The color intensity and the size of the circle are proportional to the correlation coefficients).

The correlation study of all the parameters has been given in Fig. 8. Positive correlations are indicated by blue color and negative correlations by red color. The intensity of color and size of the circle is related to correlation coefficients. A positive correlation of pendimethalin was observed with CAs, micronucleus, DNA damage, SOD and CAT enzyme activities, and lipid peroxidation whereas a negative correlation was noted with rooting percentage, root length, weight gain, MI and GR enzyme activity. Similarly, the positive correlation of curcumin with rooting percentage, root length, weight gain, MI and GR enzyme activity was recorded while a negative correlation was observed with CAs, micronucleus, DNA damage, SOD and CAT enzyme activities, and lipid peroxidation indicating its protective effect.

## Discussion

The extensive use of pesticides like pendimethalin has usually contaminated the environment. Therefore, the assessment of action mechanisms of pendimethalin and its effect on mitotic activity, growth chromosomes, genetic material and antioxidant enzymes becomes important. The protective role of curcumin, as performed in this study, is imperative in providing useful information about the recovery of injured roots.

Pendimethalin caused significant decrease in root length, rooting percentage and weight gain but curcumin at 5 mg/L and 10 mg/L showed protecting effect on these parameters when treated alone or in combination with pendimethalin. The root elongation kinetics also supported decreases in lag phase (λ), root length (A) and growth rate (µ) in pendimethalin-treated roots as well as curcumin treatment-induced recovery in µ and A. The elongation kinetics-related parameters are sensitive endpoints to assess phytotoxic responses due to physical interaction between root and pesticide. Earlier studies explained pendimethalin-induced inhibition of germination^64^, reduced seedlings^65^ and root growth^66^. The MI may be a reliable endpoint generally used for cytotoxicity measurement. The inhibition of mitotic activity described the cytotoxic potential of pendimethalin^17,67^. In this study, pendimethalin-treated roots of *A. cepa* showed MI inhibition. However, at a higher concentration of curcumin (10 mg/L), mitotic activity of the cells was improved when applied alone or in combination with pendimethalin. Several studies have been reported mitodepressive effects of herbicides^66,68–70^. Like, pendimethalin exposure inhibits cell division and disturbs the formation of microtubules in root meristems of *A. cepa*. Microtubules are important for the development of cell wall and spindle fiber leading to cell division and differentiation^71,72^.

Although various types of CAs such as fragrant, sticky chromosome, bridge, binucleated cells, unequal distribution of chromatin and vagrant chromosome were monitored in pendimethalin treated roots, at a higher concentration of curcumin (10 mg/L) reduction in CAs were observed. Stickiness is a chromatid type abnormality and is associated with the effect of pollutants on depolymerization/degradation of DNA^73^. The appearance of fragments, bridges and micronuclei indicates the clastogenic potentiality of chemicals^74,75^. The arrest of cytokinesis in the cell cycle results in binucleated cells^76^. Interference of chemicals in spindle formation may lead to chromosomal anomalies like unequal distribution of chromatin and vagrant chromosome. Similar to the results, pendimethalin induced CAs was reported by Verma and Srivastava^17^ and Ahmad et al.^67^ The findings of this study are in agreement with the reports of other pesticides including spirodiclofen^77^, diniconazole^78^, prometryne^69^ and clopyralid^79^.

Environmental contaminants can induce DNA damage that is either repaired or unrepaired. These unrepaired damages may cause alterations in DNA. Presently, the comet assay is extensively used for toxicological studies of environmental contaminants^80^. The results of the comet assay revealed that pendimethalin could induce high levels of DNA damage associated with high toxic effects in roots of *A. cepa*. However, curcumin having a protective effect when supplied with pendimethalin could reduce DNA damage. Previous studies suggested that the antioxidant system is unable to control the activities of ROS and free radicals produced during the treatment of chemicals, in turn that may induce DNA damage^81,82^. Similar response was observed in other organisms Chinese hamster over cells^83^, *Biomphalaris alexandrina* snails^84^ and freshwater fish *Clarias batrachus* L^85^.

The present study ascertained a positive correlation of antioxidant enzyme activities and DNA damage with pendimethalin. However, curcumin treatment increased antioxidant enzymes in the roots when supplied with pendimethalin indicating its protective role. Enhancement in SOD and CAT levels was observed in pendimethalin and curcumin-treated roots. Further, curcumin supply with pendimethalin reduced SOD and CAT levels. In comparison to control, these SOD and CAT levels were still higher. The increased SOD level shows the activation of the antioxidant system. SOD, CAT and GR plays important role in thwarting oxidative stress. SOD, the first line of defense against ROS, catalyzes free radicals to generate hydrogen peroxide and oxygen. Meanwhile, CAT is required to catalyze hydrogen peroxide. In addition, GR utilizes reduced glutathione to play a key role in the defense mechanism^86,87^. This study suggested a reduction in GR level pointed towards the sensitivity of the plants to pendimethalin stress. Similarly, SOD and CAT are less sensitive to pendimethalin stress in comparison to GR. Similar responses were observed under the treatment of some herbicides in hairy fleabane^88^, rice^89^, wheat and maize^86^.

The molecular docking studies showed that the 3-D structure of pendimethalin has the interactive capability to antioxidant enzymes and DNA molecules. The higher binding energy (− 8.23 kcal/mol) for CAT-complex indicated that the pendimethalin could highly perturb the 3-D structure of CAT irrespective to other SOD and GR. In contrary to molecular docking, the biochemical analysis showed that the enhanced activity of antioxidant enzymes may lead to increase more enzyme synthesis for reducing oxidative stress.

The nucleic acid interactions showed that the pendimethalin could also alter the DNA structure which was attached between adjacent DNA bases (G10-G11 and C6-A7). Such a binding mode of pendimethalin between bases of nucleic acids may lead to DNA intercalation (Snyder et al. 2004) causing genotoxicity^90,91^. It has been determined that pendimethalin selectively binds to G and C nucleotides in the B-DNA structure. The GC-rich sections of DNA can intercalate by minor groove binders and act as a DNA topoisomerase-I toxin^92^.

MDA level reflects the extent of membrane peroxidation resulting from ROS activity. The results revealed high MDA levels in roots treated with pendimethalin indicating an imbalance between ROS production and antioxidant defense leading to lipid peroxidation and oxidative stress^93^. The severity of lipid peroxidation was reduced when curcumin was supplied with pendimethalin. Similarly, exposure of watermelon to bensulfuron-methyl and penoxsulam herbicides enhanced lipid peroxidation^94^.

## Conclusion

The present study elucidates that pendimethalin induced cytotoxic and genotoxic effects, thus inhibited the growth of *A. cepa* roots. Further, pendimethalin disturbed the normal function of the cell and increased lipid peroxidation and activity of antioxidant enzymes. Molecular docking results suggested that pendimethalin could control the activity of antioxidant enzymes by interacting with their residues. However, the application of curcumin with pendimethalin alleviated the toxicity of herbicide by regulating antioxidant enzyme defense. The correlation study also suggests that curcumin can reduce chromosomal abnormalities, DNA damage, antioxidant enzyme activities (SOD and CAT) and lipid peroxidation indicating its ability to minimize oxidative stress. Hence, the synergistic use of curcumin with herbicide: pendimethalin could minimize the deteriorating impact in plants. However, a detailed study is required to unravel the exact mechanism of curcumin action in the amelioration of herbicide toxicity.
